# Supplementary material for: Synergistic Roles of Non-Homologous End Joining and Homologous Recombination in Repair of Ionizing Radiation-Induced DNA Double Strand Breaks in Mouse Embryonic Stem Cells
Source: Cells. 2024 Aug 30;13(17):1462. doi: 10.3390/cells13171462 (PMC11393957; doi:10.3390/cells13171462)
Supplement: Supplementary file 1 [file cells-13-01462-s001.zip › cells-3147924-supplementary.pdf]

cells

Supplementary Materials

(a)

Cell line	D37	Fold change compared to WT
WT	2.32	
Rad54$^{-/-}$	1.18	1.96
DNA-PKcs$^{-/-}$	0.90	2.57
DNA-PKcs$^{-/-}$ Rad54$^{-/-}$ 1	0.45	5.21
DNA-PKcs$^{-/-}$ Rad54$^{-/-}$ 2	0.37	6.28

(b)

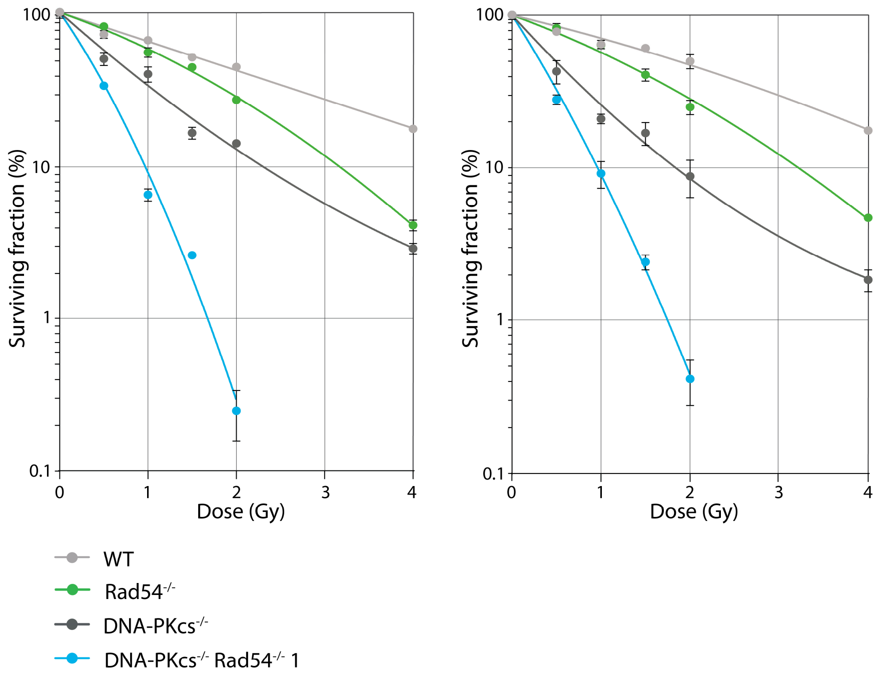

Cell line	D37	Fold change compared to WT
WT	2.28	
Rad54$^{-/-}$	1.64	1.39
DNA-PKcs$^{-/-}$	0.90	2.54
DNA-PKcs$^{-/-}$ Rad54$^{-/-}$ 1	0.46	4.91

D37	Fold change compared to WT
2.55	
1.64	1.55
0.72	3.54
0.45	5.71

Figure S1. mES DNA-PK$_{cs}^{-/-}$ Rad54$^{-/-}$ cells are hypersensitive to X-ray radiation. (**a**) Table shows D37 values and fold changes in sensitivity of clonogenic survival shown in Figure 1c. (**b**) Clonogenic survivals of mES cell lines with indicated genotypes after X-ray irradiation. Error bars represent SEM. The tables display D37 values and sensitivity fold changes of the clonogenic survivals displayed above the tables.

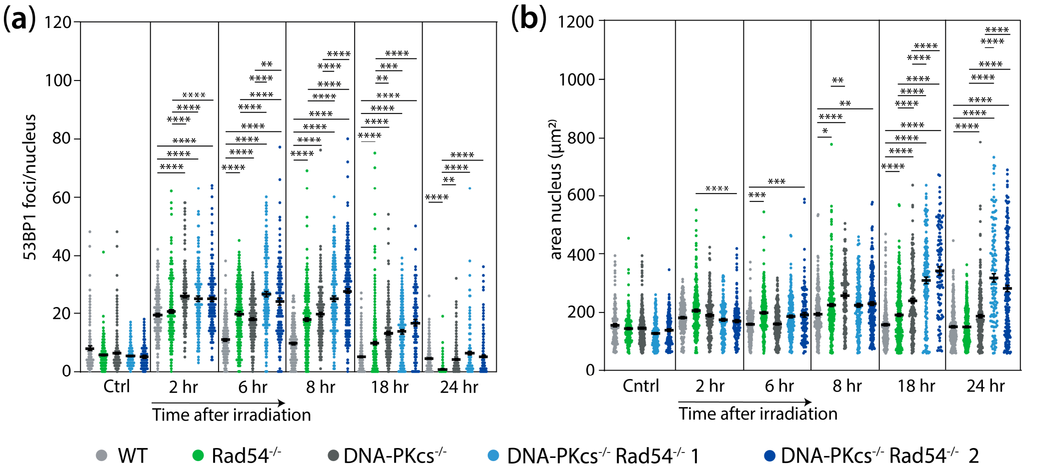

Figure S2. mES DNA-PK$_{cs}^{-/-}$ Rad54$^{-/-}$ cells show impaired 53BP1 foci resolution and increased nuclear size after 2 Gy of X-ray radiation. (**a**) Quantification of 53BP1 foci per mES nucleus. (**b**) Quantification of area of mES nuclei. Each dot represents one nucleus. Black bars indicate mean and error bars represent SEM. Asterisks represent following *p*-values: * ≤0.05; ** ≤0.01; *** ≤0.001; **** ≤0.0001.

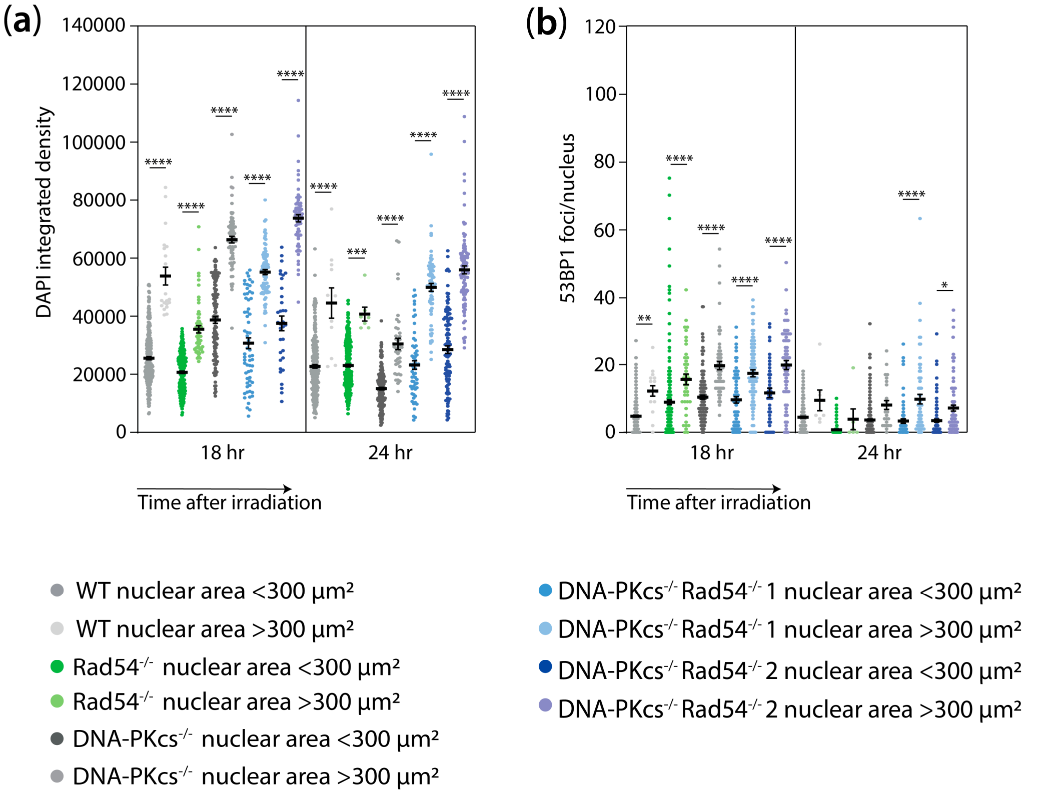

Figure S3. Cells with large nuclei (>300μm²) have increased DNA content and more DSBs. (**a**) Quantification of integrated density of DAPI signal in cells with small (<300 μm²) and large (>300 μm²) nuclei at 18 and 24 hours after X-ray irradiation. (**b**) Quantification of 53BP1 foci per nucleus in cells with small (< 300μm²) and large (> 300μm²) nuclei at 18 and 24 hours after X-ray irradiation. Each dot represents one nucleus. Black bars indicate mean and error bars represent SEM. Asterisks represent following *p*-values: * ≤0.05; ** ≤0.01; *** ≤0.001; **** ≤0.0001.

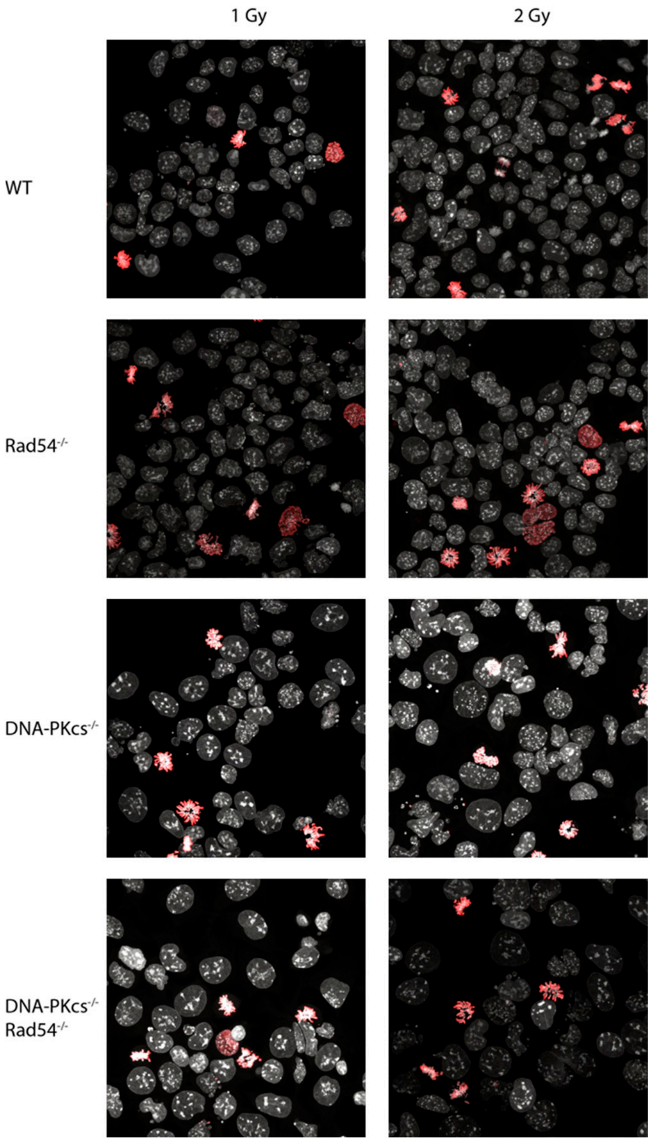

Figure S4. Phospho-H3 staining in mES cells at 24 hours after X-ray irradiation. Representative images of mES cells irradiated with 1 or 2 Gy of X-ray radiation and incubated for 24 hours. After the recovery time, cells were fixed and stained for phospho-H3. DAPI signal is shown in grey and phospho-H3 signal is shown in red.

(**a**)

Cell line	D37	Fold change compared to WT	Fold change Rad54 KO compared to Rad54 GFP-KI
WT	3.00		
Rad54$^{-/-}$	1.93	1.55	
WT Rad54$^{KR\text{-}GFP/-}$	2.49	1.21	1.29
DNA-PKcs$^{-/-}$	0.91	3.30	
DNA-PKcs$^{-/-}$ Rad54$^{WT\text{-}GFP/-}$	0.79	3.80	0.87
DNA-PKcs$^{-/-}$ Rad54$^{-/-}$ 1	0.44	6.85	
DNA-PKcs$^{-/-}$ Rad54$^{KR\text{-}GFP/-}$	0.61	4.89	1.40

(**b**)

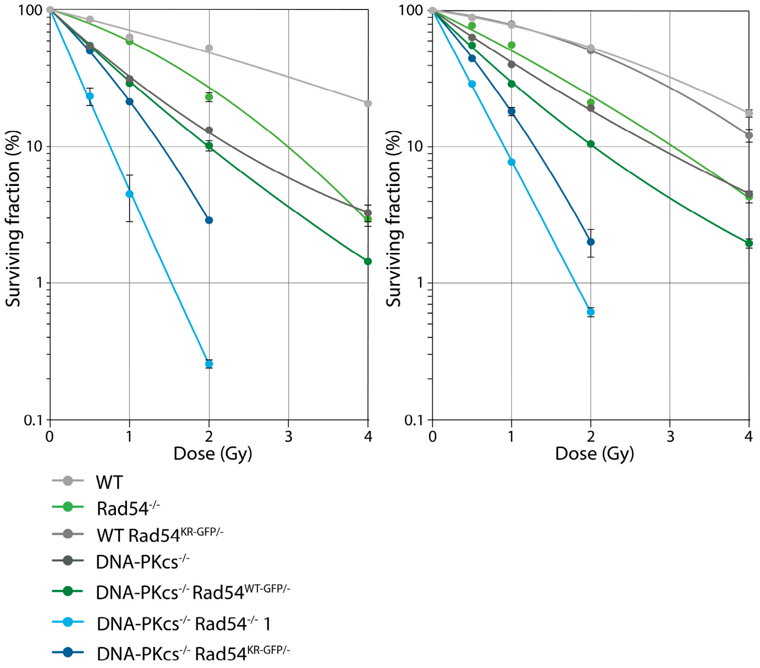

Cell line	D37	Fold change compared to WT	Fold change Rad54 KO compared to GFP-KI
WT	2.82		
Rad54$^{-/-}$	1.62	1.74	
WT Rad54$^{KR\text{-}GFP/-}$	2.52	1.12	1.56
DNA-PKcs$^{-/-}$	0.97	2.89	
DNA-PKcs$^{-/-}$ Rad54$^{WT\text{-}GFP/-}$	0.81	3.49	0.83
DNA-PKcs$^{-/-}$ Rad54$^{-/-}$ 1	0.38	7.32	
	0.64	4.43	1.65

D37	Fold change compared to WT	Fold change Rad54 KO compared to GFP-KI
2.68		
1.49	1.79	
0.88	3.05	
0.82	3.26	0.94
0.32	8.28	
0.68	3.92	2.11

Figure S5. mES DNA-PKcs$^{-/-}$ cells with ATPase defective Rad54 are hypersensitive to X-ray radiation. (**a**) Table shows D37 values and fold changes in sensitivity of clonogenic survival shown in Figure 4d. (**b**) Clonogenic survivals of mES cell lines with indicated genotypes after X-ray irradiation. Error bars represent SEM. The tables display D37 values and sensitivity fold changes of the clonogenic survivals displayed above the tables.

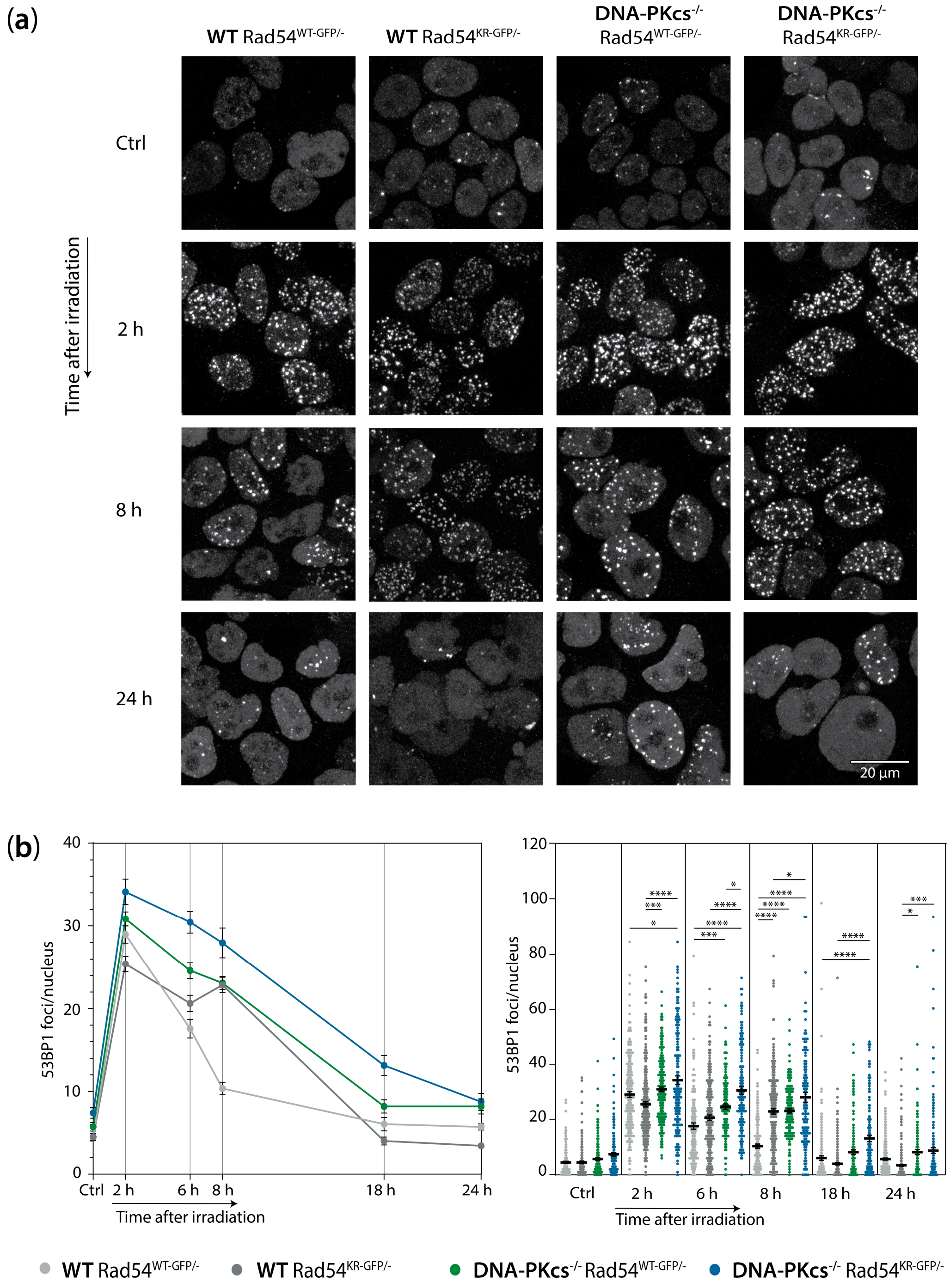

Figure S6. mES cells lacking DNA-PKcs and expressing catalytically dead Rad54 show impaired 53BP1 focus resolution. (**a**) Representative images of mES cells irradiated with 2 Gy of X-ray radiation and incubated for indicated times. After the recovery time, cells were fixated, stained and imaged for 53BP1. (**b**) Quantification of 53BP1 foci per mES nucleus. Left graph: dots indicate mean and error bars represent SEM. Right graph: each dot represents one nucleus. Black bars indicate mean and error bars represent SEM. Asterisks represent following *p*-values: * ≤0.05; *** ≤0.001; **** ≤0.0001.

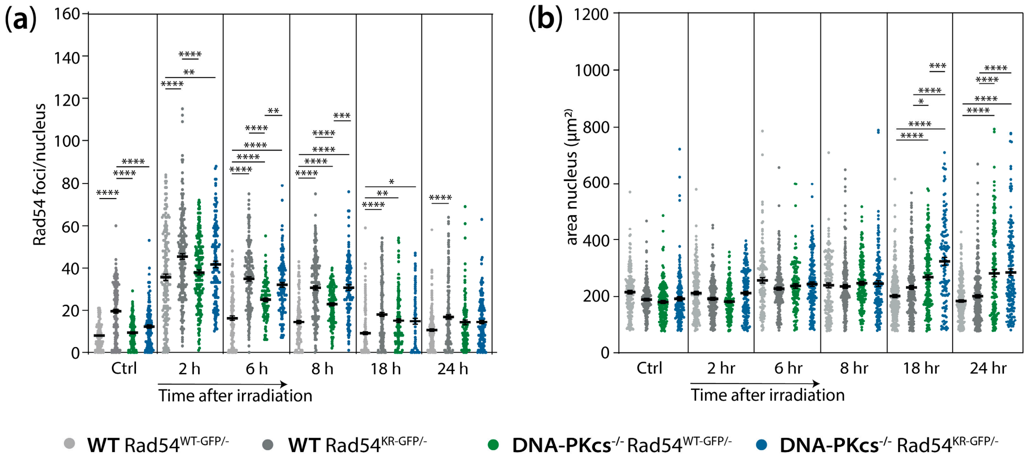

Figure S7. mES cells lacking DNA-PKcs and expressing catalytically dead Rad54 show impaired Rad54 focus resolution and increased nuclear size after 2 Gy of X-ray radiation. (a) Quantification of Rad54 foci per mES nucleus. Rad54 foci disappear in cells with enlarged nuclei (Figure 6), therefore nuclei larger than 400 μm² were excluded from the analysis. (b) Quantification of area of mES nuclei. Each dot represents one nucleus. Black bars indicate mean and error bars represent SEM. Asterisks represent following *p*-values: * ≤0.05; ** ≤0.01; *** ≤0.001; **** ≤0.0001.

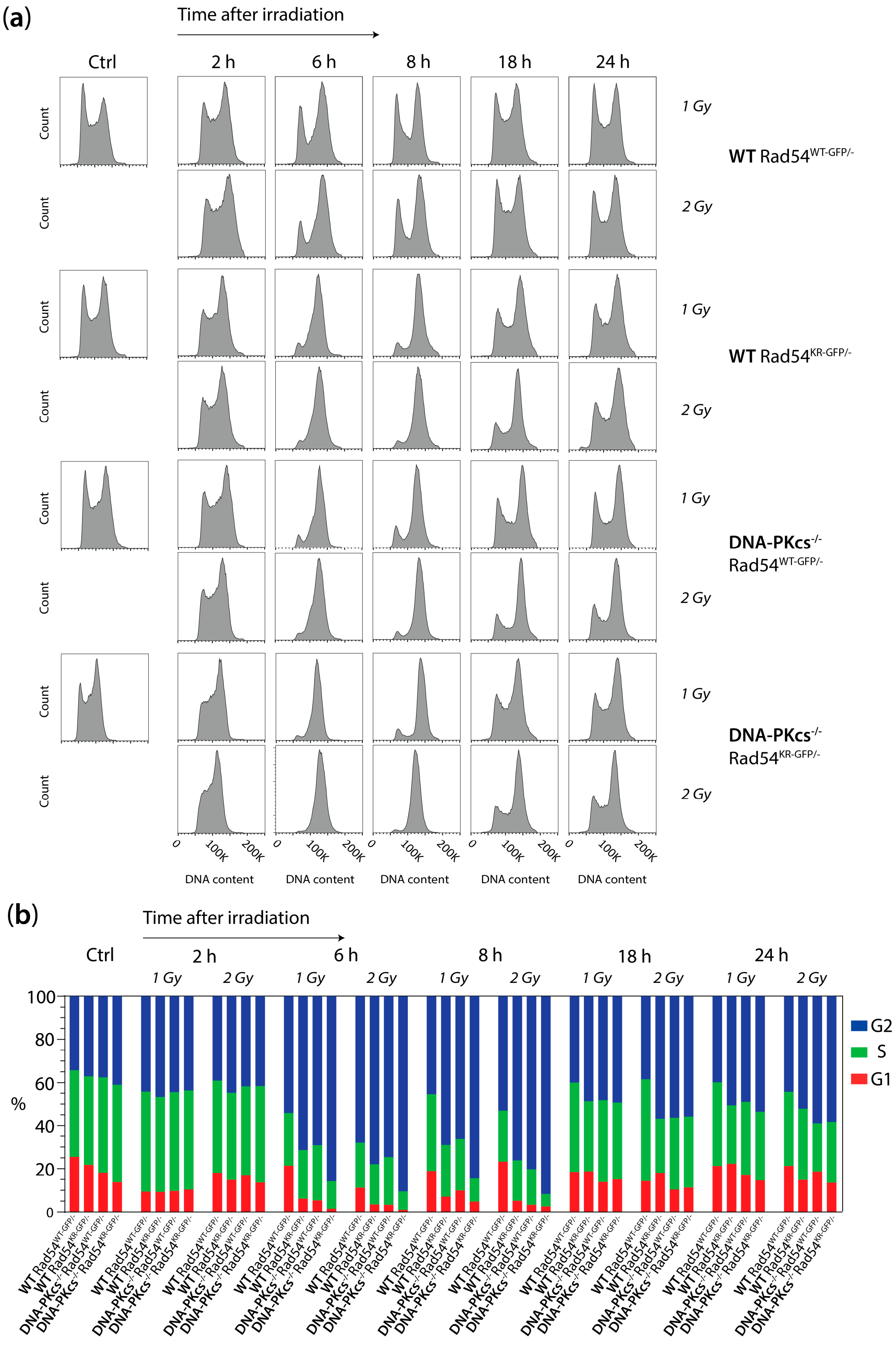

Figure S8. X-ray irradiation results in more persistent G2 phase cell cycle block in mES cells lacking DNA-PKcs and expressing catalytically dead Rad54. (**a**) mES cells were irradiated with 1 and 2 Gy of X-ray radiation and incubated for indicated times. After recovery time cells were fixed and DNA was stained using Propidium Iodide. Cell cycle distribution was analyzed using flow cytometry. (**b**) Quantification of percentage of G1, S and G2 phase cells in mES cells irradiated with 1 and 2 Gy of X-ray radiation as shown in Figure 8a.
